# Association of *HLA-A* and *HLA-B* Alleles with Lamotrigine-Induced Cutaneous Adverse Drug Reactions in the Thai Population

**DOI:** 10.3389/fphar.2017.00879

**Published:** 2017-11-29

**Authors:** Napatrupron Koomdee, Jirawat Pratoomwun, Thawinee Jantararoungtong, Voralaksana Theeramoke, Wichittra Tassaneeyakul, Jettanong Klaewsongkram, Ticha Rerkpattanapipat, Siwalee Santon, Apichaya Puangpetch, Utcharee Intusoma, Therdpong Tempark, Tayard Deesudchit, Patompong Satapornpong, Anannit Visudtibhan, Chonlaphat Sukasem

**Affiliations:** ^1^Division of Pharmacogenomics and Personalized Medicine, Department of Pathology, Faculty of Medicine, Ramathibodi Hospital, Mahidol University, Bangkok, Thailand; ^2^Laboratory for Pharmacogenomics, Somdech Phra Debaratana Medical Center, Ramathibodi Hospital, Mahidol University, Bangkok, Thailand; ^3^Manarom Hospital, Bangkok, Thailand; ^4^Department of Pharmacology, Faculty of Medicine, Khon Kaen University, Khon Kaen, Thailand; ^5^Division of Allergy and Clinical Immunology, Skin and Allergy Research Unit, Department of Medicine, Faculty of Medicine, Chulalongkorn University, Bangkok, Thailand; ^6^Division of Allergy Immunology and Rheumatology, Department of Medicine, Faculty of Medicine, Ramathibodi Hospital, Mahidol University, Bangkok, Thailand; ^7^Pediatric Neurology Unit, Department of Pediatrics, Faculty of Medicine, Prince of Songkla University, Songkhla, Thailand; ^8^Division of Pediatric Dermatology, Department of Pediatrics, Faculty of Medicine, Chulalongkorn University, Bangkok, Thailand; ^9^Division of Neurosurgery, Department of Surgery, King Chulalongkorn Memorial Hospital, Bangkok, Thailand; ^10^Division of Neurology, Department of Pediatrics, Faculty of Medicine, Ramathibodi Hospital, Mahidol University, Bangkok, Thailand; ^11^Ramathibodi Multidisciplinary Epilepsy Center, Faculty of Medicine, Ramathibodi Hospital, Mahidol University, Bangkok, Thailand

**Keywords:** lamotrigine, *HLA-A*, *HLA-B*, cutaneous adverse drug reactions, Thai population

## Abstract

**Background:** Lamotrigine (LTG) is commonly used for treatment of epilepsy and bipolar disorder. It is one of the common cause of cutaneous adverse drug reactions (CADR). Clinical symptoms of LTG-induced CADR range from maculopapular exanthema (MPE) to severe cutaneous adverse reactions (SCAR). This study aimed to determine the association of the LTG-induced CADR with human leukocyte antigen (*HLA*) alleles in Thai patients.

**Methods:** Fifteen patients with LTG-induced CADR [10 MPE; 4 Stevens–Johnson syndrome; and 1 drug reaction with eosinophilia and systemic symptoms] and 50 LTG-tolerant controls were included in the study. *HLA-A* and *HLA-B* genotyping was performed using polymerase chain reaction-sequence-specific oligonucleotides probes.

**Results:** The proportion of *HLA-A^∗^02:07* and *HLA-B^∗^15:02* allele carriers were significantly higher in the LTG-induced CADR group than in the tolerant controls [odds ratio (OR): 7.83; 95% confidence interval (CI): 1.60–38.25; *P* = 0.013, and OR: 4.89; 95% CI: 1.28–18.67; *P* = 0.014]. In addition, subjects with *HLA-A^∗^33:03*, *HLA-B^∗^15:02*, and *HLA-B^∗^44:03* were significantly higher in the LTG-induced MPE group than in the tolerant controls (OR: 8.27; 95% CI: 1.83–37.41; *P* = 0.005, OR: 7.33; 95% CI: 1.63–33.02; *P* = 0.005; and OR: 10.29; 95% CI: 1.45–72.81; *P* = 0.029). In contrast to the LTG-induced MPE group, there were no significant differences between *HLA* alleles and LTG-induced SCAR group.

**Conclusion:** *HLA-A^∗^02:07* and *HLA-B^∗^15:02* were associated with LTG-induced CADR in Thai patients. We also identified an association between *HLA-A^∗^33:03*, *HLA-B^∗^15:02*, and *HLA-B^∗^44:03* and LTG-induced MPE in this population. These results suggest that these alleles could be useful screening markers for preventing CADR before LTG treatment in Thai patients, but further replication studies with larger sample sizes are needed.

## Introduction

Lamotrigine (LTG) is a phenyltriazine derivative which is used in the treatment of epilepsy and bipolar disorder. It is one of the aromatic antiepileptic drugs (AEDs) which together are the most common cause of cutaneous adverse reactions (CADR) (Arif et al., 2007). The current widely used AEDs include carbamazepine (CBZ), oxcarbazepine (OXC), phenytoin (PHT), and phenobarbital (PB) (Maggs et al., 2000; Arif et al., 2007; Chung et al., 2010). CADR manifestations range from mild maculopapular exanthema (MPE) to severe cutaneous adverse reactions (SCAR), including Stevens–Johnson syndrome (SJS), toxic epidermal necrolysis (TEN), and drug reactions with eosinophilia and systemic symptoms (DRESS). The mortality rates are 1–5% of patients with AED-induced SJS and up to 30% in AED-induced TEN (Roujeau and Stern, 1994; Harr and French, 2010; Yang et al., 2011). The incidences of SJS and TEN range between 1 and 10 cases per 10,000 patients (Mockenhaupt et al., 2005).

Pharmacogenetic studies have identified genetic associations between the human leukocyte antigen (*HLA*) allele and AEDs-induced CADR. According to specific AEDs medication, AEDs-induced SJS/TEN has been associated with specific *HLA* alleles in various populations, namely *HLA-B^∗^15:02* and CBZ in the Han Chinese and Thai populations (Chung et al., 2004; Lim et al., 2008; Locharernkul et al., 2008; Tassaneeyakul et al., 2010) but not in the Japanese (Kaniwa et al., 2008) and European population (Alfirevic et al., 2006), *HLA-B^∗^15:02* and OXC in Chinese and Thai populations (Chen et al., 2017). LTG is the most common AED used in Thailand. It has a similar chemical structure to CBZ, and high cross-reactivity rates of skin reaction from the group of AEDs in Han Chinese epilepsy patients have been reported (Greenwood, 2000; Maggs et al., 2000; Wang et al., 2010). LTG-induced SJS/TEN has been associated with *HLA-B^∗^44:03* in Korean patients but no association was found with *HLA-B^∗^15:02* (An et al., 2010; Hung et al., 2010; Shi et al., 2011; Park et al., 2015). In addition, *HLA-A^∗^30:01* and *HLA-B^∗^13:02* have been associated with a higher risk of LTG-induced MPE in Han Chinese (Li et al., 2013). However, there are no specific *HLA* alleles associated with LTG-induced CADR and an association between LTG-induced CADR and *HLA* alleles in Thailand has not been identified. Therefore, we aimed to examine the association between *HLA-A* and *HLA-B* and LTG-induced CADR in the Thai population.

## Materials and Methods

### Subjects

A case–control study was performed at the Laboratory for Pharmacogenomics, Somdech Phra Debaratana Medical Center (SDMC), Ramathibodi Hospital, Thailand. Fifteen LTG-induced CADR (4 cases of SJS, 1 case of DRESS, and 10 cases of MPE) were recruited from the Faculty of Medicine, Ramathibodi Hospital, Mahidol University, Manarom Hospital, and Srinagarind Hospital between 2011 and 2015. All patients who developed CADR, as SJS, MPE, or DRESS, within 2 months after initiating LTG treatment were recruited for the study. The dermatological diagnosis was made by a dermatologist or allergist who reviewed photographs, pathological slides, clinical morphology, and medical records. MPE was defined as the presence of erythematous macules and papules without mucosal involvement, and in which the skin rash resolved after the drug was discontinued. The RegiSCAR criteria were used to establish SJS and DRESS. SJS was defined as skin detachment of BSA <10%, and TEN as skin detachment of BSA >30%. DRESS was defined as presenting with fever, maculopapular rash with internal organ involvement, and hematologic abnormalities. Patients who had been taking LTG for more than 6 months without evidence of cutaneous adverse effects were recruited as LTG-tolerant controls. In addition, the general population who had not taken LTG and had no history of drug-induced CADR were included in this study. Both case and control subjects were independently recruited with no family relationship. Data for this general control group were obtained from 369 and 986 subjects undergoing *HLA-A* and *-B* genotyping, respectively.

The study was approved by the Ramathibodi Hospital Ethical Review Board, and informed, written consent was obtained from all participants.

### *HLA* Genotyping

Genomic DNA samples were extracted from EDTA blood using a MagNA pure compact Nucleic Acid Isolation Kit on a MagNApure Compact machine. *HLA* alleles were genotyped using polymerase chain reaction-sequence-specific oligonucleotides (PCR-SSOs) according to the manufacturer’s protocol. In brief, diluted DNA samples were amplified by PCR using a GeneAmp^®^PCR System 9700 (Applied Biosystems, Waltham, MA, United States). The PCR products were then hybridized against a panel of oligonucleotide probes on coated polystyrene microspheres that had sequences complementary to stretches of polymorphisms within the target *HLA-A*, *B* alleles (a LABType^®^SSO, One Lambda Inc. Kit). The amplicon–probe complex was visualized using a colorimetric reaction and fluorescence detection technology (Luminex^®^IS 100). Data analysis for the *HLA* assays was performed with the software package HLA fusion 2.0.

### Statistical Analysis

Statistical analyses were performed using SPSS for Windows (version 16.0; SPSS, Chicago, IL, United States). Means and standard deviations were calculated for continuous variables. Dosages of LTG intake were described as median and interquartile range (IQR). To detect differences in the clinical characteristics between the case and control groups, an independent *t-*test was used for continuous variables. Chi-square test and Fisher’s exact test were used to describe the differences in frequencies of the *HLA-A*, *B* alleles between the groups. Haplotype association analysis was carried out using the “haplo.stats” package. The level of statistical significance was set at *p* < 0.05 (two-sided).

## Results

### Patients and Characteristics

DNA samples from 15 LTG-induced CADR patients (10 cases with MPE, 4 cases with SJS, and 1 case with DRESS) and 50 LTG-tolerant controls and the general population group were genotyped. The mean age of the LTG-induced CADR patients was 35.2 ± 22.1 and 73.3% were female. The median LTG dosage was 50 mg per day. There were no significant differences in gender, age, dosage of LTG, and concomitant use of valproic acid between the LTG-induced CADR patients and the LTG-tolerant patients (**Table 1**).

**Table 1:** Clinical characteristics of patients in the lamotrigine (LTG)-induced cutaneous adverse drug reactions group and the LTG-tolerant group.

	LTG-induced CADR group (*n* = 15)	LTG-tolerant group (*n* = 50)	*P*-value
Gender, *n*(%)			1.000
Male	4 (26.7)	12 (24.0)	
Female	11 (73.3)	38 (76.0)	
Mean age, mean ± *SD* (years)	35.2 ± 22.1	38.2 ± 19.0	0.203
Dosage of LTG, median (IQR) (mg/day)	50 (25–100)	100 (25–100)	0.208
Indication for LTG, *n*(%)			0.290
Epilepsy	6 (40.0)	20 (40.0)	
Bipolar disorder	5 (33.3)	8 (16.0)	
Depressive disorder	3 (20.0)	6 (12.0)	
Major depressive disorder	1 (6.7)	3 (6.0)	
Mood stabilizer	0 (0)	4 (8.0)	
Others	0 (0)	9 (18.0)	
Concomitant use of valproic acid, *n*(%)	1 (6.7)	4 (8.0)	1.000


*CADR, cutaneous adverse drug reaction; LTG, lamotrigine.*

### The *HLA* Allele and LTG-Induced CADR

The *HLA-A* and *HLA-B* genotypes of the LTG-induced CADR patients are shown in **Table 2**. The *HLA* genotypes for the treatment tolerant controls and the comparison of the *HLA* allele found in the CADR patients and control groups (LTG-tolerant controls and general population) are shown in Supplementary Tables 1, 2. The *HLA* alleles that showed a significant association when compared with the tolerant controls and general population are presented in **Table 3**. We found the *HLA-B^∗^15:02* allele in 40.0% of patients who developed CADR and in 12.0% of the tolerant patients. The proportion of patients carrying the *HLA-B^∗^15:02* allele was significantly higher in LTG-induced CADR cases than in both the treatment controls and general population groups with odds ratios (OR) of 4.89, 95% CI = 1.28–18.66, *P*-value = 0.014 and OR = 3.63, 95% CI = 1.27–10.34, *P*-value = 0.027, respectively. In addition, we also found a significant association between LTG-induced CADR patients and both *HLA-B^∗^35:08* and *HLA-B^∗^39:01* when compared with the general population with OR = 70.36, 95% CI = 4.19–1182.21, *P*-value = 0.030 and OR = 10.68, 95% CI = 2.20–51.83, *P*-value = 0.022, respectively.

**Table 2:** *HLA-A* and *HLA-B* genotype data of LTG-induced cutaneous adverse drug reaction patients.

No.	Sex	Phenotype	*HLA-A* genotype	*HLA-B* genotype
1	F	MPE	0207/3303	1502/4403
2	F	MPE	0207/3303	4601/5801
3	M	MPE	2402/3303	1513/4403
4	F	MPE	0206/1101	1502/5101
5	F	MPE	1101/1101	4001/4601
6	F	MPE	1101/3001	1502/5201
7	F	MPE	2402/3303	1301/5801
8	M	MPE	0201/3303	1502/4403
9	M	MPE	0207/3303	1502/3508
10	F	MPE	1101/3303	3901/5801
11	F	SJS	0207/0207	4601/4601
12	F	SJS	1101/2402	1535/1802
13	F	SJS	1101/1101	3901/4001
14	F	SJS	0207/2402	1301/1502
15	M	DRESS	1101/1102	4601/5502


*DRESS, drug reaction with eosinophilia and systemic symptoms; F, female; *HLA*, human leukocyte antigen; M, male; MPE, maculopapular exanthema; SJS, Stevens–Johnson syndrome.*

**Table 3:** List of *HLA* alleles that showed a significant association with LTG-induced cutaneous adverse drug reactions.

HLA allele	LTG-induced CADR +/total	LTG-tolerant +/total	Cases versus LTG-tolerant		General population +/total	Cases versus general population	
					
			OR (95% CI)	*P*-value		OR (95% CI)	*P*-value
***HLA-A^∗^02:07***	**5/15**	**3/50**	**7.83 (1.60–38.25)**	**0.013**	**49/369**	**3.27 (1.07–9.96)**	**0.029**
***HLA-A^∗^33:03***	7/15	11/50	3.10 (0.92–10.46)	0.061	**80/369**	**3.16 (1.11–8.98)**	**0.023**
***HLA-B^∗^15:02***	**6/15**	**6/50**	**4.89 (1.28–18.66)**	**0.014**	**153/986**	**3.63 (1.27–10.34)**	**0.027**
***HLA-B^∗^35:08***	1/15	0/50	10.45 (0.40–270.41)	0.231	**1/986**	**70.36 (4.19–1182.21)**	**0.030**
***HLA-B^∗^39:01***	2/15	1/50	7.54 (0.63–89.76)	0.131	**14/986**	**10.68 (2.20–51.83)**	**0.022**


*CI, confidence interval; CADR, cutaneous adverse drug reaction; *HLA*, human leukocyte antigen; LTG, lamotrigine; OR, odds ratio; +/total, number of subjects positive for HLA allele/number of total subjects included in the study. *P*-values were calculated by Fisher’s exact test. Statistically significant values are highlighted in bold.*

In subgroup analysis of LTG-induced CADR, a significant association between LTG-induced MPE and *HLA-B^∗^15:02* was found when compared with the tolerant controls and general population (OR = 7.33, 95% CI = 1.63–33.02, *P*-value = 0.005 and OR = 5.44, 95% CI = 1.56–19.03, *P*-value = 0.003, respectively). Interestingly, a significant association between LTG-induced MPE and *HLA-B^∗^44:03* was found when compared with both control groups (OR = 10.29, 95% CI = 1.45–72.81, *P*-value = 0.029 and OR = 4.73, 95% CI = 1.20–18.62, *P*-value = 0.046, respectively), whereas *HLA-B^∗^35:08* was significantly associated only with the general population (OR = 109.44, 95% CI = 6.34–1889.11, *P*-value = 0.020) (**Table 4**); nevertheless, no significant associations were found in LTG-induced SCAR.

**Table 4:** The associations of individual *HLA* alleles with LTG-induced cutaneous adverse drug reactions among the different subgroups.

	HLA allele	LTG-induced CADR +/total	LTG-tolerant +/total	Cases versus LTG-tolerant		General population +/total	Cases versus general population	
						
				OR (95% CI)	*P*-value		OR (95% CI)	*P*-value
MPE	*HLA-A^∗^02:07*	3/10	3/50	6.71 (1.13–40.07)	0.052	49/369	2.80 (0.70–11.19)	0.145
	***HLA-A^∗^33:03***	**7/10**	**11/50**	**8.27 (1.83–37.41)**	**0.005**	**80/369**	**8.43 (2.13–33.34)**	**0.002**
	***HLA-B^∗^15:02***	**5/10**	**6/50**	**7.33 (1.63–33.02)**	**0.005**	**153/986**	**5.44 (1.56–19.03)**	**0.003**
	***HLA-B^∗^35:08***	1/10	0/50	15.95 (0.60–421.64)	0.167	**1/986**	**109.44 (6.34–1889.11)**	**0.020**
	*HLA-B^∗^39:01*	1/10	1/50	3.44 (0.31–95.21)	0.308	14/986	7.71 (0.92–65.06)	0.141
	***HLA-B^∗^44:03***	**3/10**	**2/50**	**10.29 (1.45–72.81)**	**0.029**	**82/986**	**4.73 (1.20–18.62)**	**0.046**
SCAR	*HLA-A^∗^02:07*	2/5	3/50	10.44 (1.23–88.44)	0.060	49/369	4.35 (0.70–26.72)	0.139
	*HLA-A^∗^33:03*	0/5	11/50	0.31 (0.02–6.08)	0.570	80/369	0.33 (0.02–5.98)	0.589
	*HLA-B^∗^15:02*	1/5	6/50	1.83 (0.18–19.25)	0.508	153/986	0.74 (0.08–6.62)	0.571
	*HLA-B^∗^35:08*	0/5	0/50	NA	NA	1/986	59.73 (2.18–1633.30)	1.000
	*HLA-B^∗^39:01*	1/5	1/50	12.25 (0.64–234.81)	0.175	14/986	0.06 (0.01–0.55)	0.074


*CI, confidence interval; CADR, cutaneous adverse drug reaction; *HLA*, human leukocyte antigen; LTG, lamotrigine; MPE, maculopapular exanthema; NA, not available; OR, odds ratio; SCAR, severe cutaneous adverse reaction; +/total, number of subjects positive for HLA allele/number of total subjects included in the study. *P*-values were calculated by Fisher’s exact test. Statistically significant values are highlighted in bold.*

Compared with the *HLA-B* allele, *HLA-A^∗^02:07* was present in 33.3% of LTG-induced CADR patients and showed significantly higher frequencies than both the treatment control and general population groups with OR = 7.83, 95% CI = 1.60–38.25, *P*-value = 0.013 and OR = 3.27, 95% CI = 1.07–9.96, *P*-value = 0.029, respectively; in addition, *HLA-A^∗^33:03* also had a significantly higher frequency than in the general population (OR = 3.16, 95% CI = 1.11–8.98, *P*-value = 0.023). Moreover, we found a significant association of LTG-induced MPE with *HLA-A^∗^33:03* compared with the tolerant controls group (OR = 8.27, 95% CI = 1.83–37.41, *P*-value = 0.005) and general population group (OR = 8.43, 95% CI = 2.13–33.34, *P*-value = 0.002) as shown in **Table 4**.

The analysis of *HLA-A* and *HLA-B* haplotypes have demonstrated that the *HLA-A^∗^02:07/HLA-B^∗^15:02* and *HLA-A^∗^33:03/HLA-B^∗^15:02* haplotypes were not found to be associated with LTG-induced CADR (data not shown).

## Discussion

In the present study, we found the significant association between LTG-induced CADR and *HLA-A^∗^02:07* and *HLA-B^∗^15:02* when compared with both tolerant and general population controls. In addition, the *HLA-A^∗^33:03* allele was present at a significantly higher rate in LTG-induced CADR patients than in the general population controls. These results suggest that *HLA-B^∗^15:02*, alone, might not be the only risk factor for LTG-induced CADR, but *HLA-A^∗^02:07* and *HLA-A^∗^33:03* may also be risk factors for LTG-induced CADR. The subgroup analysis revealed that the proportion of patients carrying the *HLA-B^∗^15:02* allele was significantly higher in LTG-induced MPE cases than in both the tolerant control and general population groups, which is very different from previous studies in which *HLA-B^∗^15:02* was not found to be associated with LTG-induced MPE (An et al., 2010; Shi et al., 2011). We demonstrated that *HLA-A^∗^33:03* may be a risk factors for LTG-induced MPE, which again is different from the findings of a previous study in the Han Chinese population which found that patients carrying *HLA-A^∗^33:03* had a lower risk for LTG-induced MPE (Li et al., 2013). The same study also found that patients carrying either of the HLA*-A^∗^30:01* or *HLA-B^∗^13:02* alleles had increased risk for LTG-induced MPE (Li et al., 2013). In this study, we did not find a significant association between *HLA-A^∗^30:01* or *HLA-B^∗^13:02* and LTG-induced MPE. One study found the *HLA-A^∗^30:01* allele in 1 of 10 LTG-induced MPE patients, while *HLA-B^∗^13:02* was absent in the same 10 LTG-induced MPE patients, which the authors suggested could be because of the low frequency of *HLA-B^∗^13:02* (1.4%) in the Thai population (Puangpetch et al., 2015).

Conversely, no significant association between LTG-induced SCAR and *HLA-B^∗^15:02* was found when compared with the two control groups, nor did we find significant differences in the other *HLA* alleles between the LTG-induced SCAR group and the two control groups. Earlier studies on CBZ-induced SJS/TEN and the *HLA-B^∗^15:02* allele reported no associations with the MPE group (Chung et al., 2004; Hung et al., 2006). The chemical structure of LTG includes aromatic rings similar to CBZ and shared a common risk allele causing SJS/TEN which similarities with other aromatic AEDs, namely PHT and OXC (Hung et al., 2010). Previous studies have found an association between LTG-induced SJS/TEN and *HLA-B^∗^15:02* in Han Chinese (An et al., 2010; Hung et al., 2010), but other studies have found no association in Japanese patients (Kaniwa et al., 2008) or European population (Alfirevic et al., 2006), due to the fact that the *HLA-B^∗^15:02* allele is rare in the Japanese population (0.1%) and people of European descent (0%), according to data from Lee et al. (2010). However, data from all of these studies were limited due to the small sample sizes of LTG-induced SJS patients; therefore, association studies between the *HLA* genotype and LTG-induced SJS could not be performed.

In this study, we report for the first time a significant association between *HLA-B^∗^35:08* and LTG-induced CADR or MPE, although this allele has been reported in only one case of LTG-induced MPE and once in the general population, as a result of this allele being very rare in the Thai population (less than 1%, data from Puangpetch et al., 2015). The interpretation of data from studies with a small sample size can only be tentative, and further investigations with larger sample sizes are needed. Similarly to *HLA-B^∗^35:08*, the association of LTG-induced MPE and *HLA-B^∗^44:03* alleles was firstly reported in the Thai population. One recent study from Korea found that *HLA-B^∗^44:03* was associated with LTG-induced SJS/TEN (OR: 12.75, 95% CI: 1.03–157.14, and *P*-value = 0.053) (Park et al., 2016).

One study found that LTG-induced CADR in the Japanese population was associated with *HLA* class II alleles, including *HLA-DRB1^∗^04:05*, *HLA-DQB1^∗^04:01*, and *HLA-DQA1^∗^03:03* (Ito et al., 2015), but *HLA* class II genotyping was not performed in this current study. However, it would be interesting to investigate the association of *HLA* class II and LTG-induced CADR in each population and with a large number of patients to better understand any association. Nevertheless, previous studies identified age and concomitant use of LTG and valproic acid as risk factors for LTG-induced CADR (Cheung et al., 2013; Egunsola et al., 2015). However, in our study we did not find that age and concomitant therapy with valproic acid were risk factors for LTG-induced CADR.

Apart from the *HLA* alleles, drug-metabolizing enzymes may be a risk factor for developing CADR. LTG is primarily metabolized by uridine diphosphate glucuronosyltransferases (UGT), including UGT1A4 and UGT2B7 (Perucca, 2006). 2-*N*-Glucuronide conjugates are the major inactive metabolite of LTG and elimination from the body by any enzyme variant either than one of these UGT enzymes will affect the risk of cutaneous adverse drug reactions (Rowland et al., 2006). A recent study on drug metabolizing enzymes found that the *cytochrome P4502C9* (CYP2C9) influenced PHT-induced SCAR in the Thai population (Tassaneeyakul et al., 2016). Further association studies are required to determine the association between the glucuronidation metabolic pathway and LTG-induced CADR.

## Conclusion

We found a statistically significant association of the *HLA-A^∗^02:07* and *HLA-B^∗^15:02* alleles with LTG-induced CADR in the Thai population. Therefore, these two alleles might be potential risk markers for LTG-induced CADR in Thailand. To confirm these findings, further large-scale studies are required.

## Ethics Statement

The project has been reviewed and approved by the Committee on Human Right Related to Research Involving Human Subjects, based on the Declaration of Helsinki (MURA2012/307/S2, June 16).

## Author Contributions

CS, WT, and NK designed the research study. TR, TT, and JK diagnosed and recruited the subjects. VT, AP, and TD collected the clinical data; NK, TJ, SS, UI, and AV performed genotyping and evaluated the results. JP and PS analyzed the data. CS, JP, and PS wrote the manuscript. CS and AP reviewed and edited the manuscript.

## Conflict of Interest Statement

The authors declare that the research was conducted in the absence of any commercial or financial relationships that could be construed as a potential conflict of interest. The reviewer ZR-P and handling editor declared their shared affiliation.
