# Supplementary material for: Association of HLA-A and HLA-B Alleles with Lamotrigine-Induced Cutaneous Adverse Drug Reactions in the Thai Population
Source: Front Pharmacol. 2017 Nov 29;8:879. doi: 10.3389/fphar.2017.00879 (PMC5712579; doi:10.3389/fphar.2017.00879)
Supplement: Supplementary file 1 [file Table_1.doc]

**Supplementary Table 1** *HLA-A* and *HLA-B* genotype data of lamotrigine-tolerant controls

| No. | Sex | *HLA-A* genotype | *HLA-B* genotype |
| --- | --- | --- | --- |
| 1 | M | 0206/2402 | 4001/4803 |
| 2 | F | 0201/1101 | 4001/4601 |
| 3 | F | 0207/2402 | 4601/4601 |
| 4 | F | 2402/2402 | 1502/4403 |
| 5 | F | 1101/3001 | 1302/4601 |
| 6 | F | 2601/3401 | 0801/1521 |
| 7 | F | 0203/1101 | 4001/4601 |
| 8 | M | 0201/1101 | 1301/5701 |
| 9 | F | 0211/1101 | 1301/4601 |
| 10 | F | 2402/2401 | 2706/4601 |
| 11 | M | 1101/1101 | 1801/5602 |
| 12 | F | 1102/3303 | 1502/1525 |
| 13 | F | 1101/1101 | 0705/5601 |
| 14 | F | 0201/0203 | 1517/4001 |
| 15 | F | 3303/3303 | 1502/4403 |
| 16 | F | 1101/1102 | 1527/5401 |
| 17 | F | 1101/3303 | 4001/5801 |
| 18 | F | 0301/3101 | 3503/3802 |
| 19 | M | 1101/3101 | 1535/3501 |
| 20 | F | 1101/1102 | 2704/4801 |
| 21 | F | 0203/2402 | 3503/4001 |
| 22 | F | 3001/3303 | 1302/1502 |
| 23 | F | 1101/2402 | 1301/5101 |
| 24 | F | 2402/2402 | 4001/4001 |
| 25 | M | 2402/3303 | 1501/5801 |
| 26 | F | 0203/1103 | 5502/5801 |
| 27 | F | 0101/3303 | 5201/5801 |
| 28 | F | 2402/3303 | 4002/5401 |
| 29 | F | 0301/2407 | 3501/5101 |
| 30 | F | 0201/1101 | 1518/5502 |
| 31 | M | 0206/3303 | 1301/3909 |
| 32 | F | 1101/2601 | 1518/3901 |
| 33 | F | 3301/3301 | 3503/4601 |
| 34 | M | 2402/2402 | 4601/5601 |
| 35 | M | 1101/3101 | 1301/4001 |
| 36 | F | 0201/1101 | 3701/4001 |
| 37 | M | 1101/2402 | 1301/4001 |
| 38 | M | 0207/2410 | 1802/1802 |
| 39 | F | 0206/1102 | 2704/5501 |
| 40 | F | 0207/3303 | 1502/4601 |
| 41 | F | 0203/3303 | 5502/5801 |
| 42 | F | 0201/3303 | 1518/4402 |
| 43 | F | 2402/3108 | 4801/5512 |
| 44 | F | 1101/3401 | 1502/1521 |
| 45 | F | 2402/2410 | 3505/4601 |
| 46 | F | 0201/2402 | 4601/4601 |
| 47 | M | 2402/2402 | 4001/5401 |
| 48 | F | 1101/2407 | 1801/5502 |
| 49 | M | 0201/1101 | 1301/1511 |
| 50 | F | 0201/2402 | 2706/4001 |

F, female; M, male; *HLA*, human leukocyte antigen

**Supplementary Table 2** Associations of individual *HLA* alleles with lamotrigine-induced cutaneous adverse drug reactions.

|  | | *HLA allele* | LTG-induced CADR  +/total | LTG-tolerant  +/total | Cases versus LTG-tolerant | | General population  +/total | Cases versus general population | |
| --- | --- | --- | --- | --- | --- | --- | --- | --- | --- |
|  |  |  |  |  | OR (95% CI) | P-value |  | OR (95% CI) | P-value |
| CADR | | *HLA-A*02:01* | 1/15 | 9/50 | 0.33 (0.04-2.80) | 0.431 | 39/369 | 0.60 (0.08-4.72) | 1.000 |
|  | | *HLA-A*02:06* | 1/15 | 3/50 | 1.12 (0.11-11.62) | 1.000 | 15/369 | 1.69 (0.21-13.68) | 0.478 |
|  | | ***HLA-A*02:07*** | **5/15** | **3/50** | **7.83 (1.60-38.25)** | **0.013** | **49/369** | **3.27 (1.07-9.96)** | **0.029** |
|  | | *HLA-A*11:01* | 7/15 | 20/50 | 1.31 (0.41-4.19) | 0.767 | 152/369 | 1.25 (0.44-3.52) | 0.673 |
|  | | *HLA-A*11:02* | 1/15 | 4/50 | 0.82 (0.09-7.96) | 1.000 | 11/369 | 2.33 (0.28-19.28) | 0.384 |
|  | | *HLA-A*24:02* | 4/15 | 16/50 | 0.77 (0.21-2.81) | 0.761 | 80/369 | 1.31 (0.41-4.24) | 0.749 |
|  | | *HLA-A*30:01* | 1/15 | 2/50 | 1.71 (0.15-20.33) | 0.551 | 16/369 | 1.58 (0.20-12.74) | 0.500 |
|  | | ***HLA-A*33:03*** | 7/15 | 11/50 | 3.10 (0.92-10.46) | 0.061 | **80/369** | **3.16 (1.11-8.98)** | **0.023** |
|  | | *HLA-B*13:01* | 2/15 | 7/50 | 0.95 (0.18-5.12) | 1.000 | 131/986 | 1.00 (0.22-4.50) | 1.000 |
|  | *HLA-B*13:02* | | 0/15 | 2/50 | 0.63 (0.03-13.75) | 1.000 | 27/986 | 1.13 (0.07-19.30) | 1.000 |
|  | ***HLA-B*15:02*** | | **6/15** | **6/50** | **4.89 (1.28-18.66)** | **0.014** | **153/986** | **3.63 (1.27-10.34)** | **0.027** |
|  | *HLA-B*15:13* | | 1/15 | 0/50 | 10.45 (0.40-270.41) | 0.231 | 11/986 | 6.33 (0.77-52.43) | 0.167 |
|  | *HLA-B*15:35* | | 1/15 | 1/50 | 3.50 (0.21-59.59) | 0.411 | 16/986 | 4.33 (0.54-34.94) | 0.228 |
|  | *HLA-B*18:02* | | 1/15 | 1/50 | 3.50 (0.21-59.59) | 0.411 | 46/986 | 1.46 (0.19-11.34) | 0.516 |

|  | ***HLA-B*35:08*** | 1/15 | 0/50 | 10.45 (0.40-270.41) | 0.231 | **1/986** | **70.36 (4.19-1182.21)** | **0.030** |
| --- | --- | --- | --- | --- | --- | --- | --- | --- |
|  | ***HLA-B*39:01*** | 2/15 | 1/50 | 7.54 (0.63-89.76) | 0.131 | **14/986** | **10.68 (2.20-51.83)** | **0.022** |
|  | *HLA-B*40:01* | 2/15 | 11/50 | 0.55 (0.11-2.79) | 0.715 | 156/986 | 0.82 (0.18-3.66) | 1.000 |
|  | *HLA-B*44:03* | 3/15 | 2/50 | 6.00 (0.90-40.02) | 0.076 | 82/986 | 2.76 (0.76-9.96) | 0.128 |
|  | *HLA-B*46:01* | 4/15 | 11/50 | 1.29 (0.34-4.85) | 0.734 | 207/986 | 1.37 (0.43-4.34) | 0.534 |
|  | *HLA-B*51:01* | 1/15 | 1/50 | 3.50 (0.21-59.59) | 0.411 | 65/986 | 1.01 (0.13-7.82) | 1.000 |
|  | *HLA-B*52:01* | 1/15 | 1/50 | 3.50 (0.21-59.59) | 0.411 | 43/986 | 1.57 (0.20-12.19) | 0.493 |
|  | *HLA-B*55:02* | 1/15 | 4/50 | 0.82 (0.08-7.96) | 1.000 | 23/986 | 2.99 (0.38-23.71) | 0.307 |
|  | *HLA-B*58:01* | 3/15 | 5/50 | 2.25 (0.47-10.78) | 0.373 | 161/986 | 1.28 (0.36-4.59) | 0.723 |
| MPE | *HLA-A*02:01* | 1/10 | 9/50 | 0.51 (0.06-4.52) | 1.000 | 39/369 | 0.94 (0.12-7.62) | 1.000 |
|  | *HLA-A*02:06* | 1/10 | 3/50 | 1.74 (0.16-18.68) | 0.528 | 15/369 | 2.62 (0.31-22.06) | 0.354 |
|  | *HLA-A*02:07* | 3/10 | 3/50 | 6.71 (1.13-40.07) | 0.052 | 49/369 | 2.80 (0.70-11.19) | 0.145 |
|  | *HLA-A*11:01* | 4/10 | 20/50 | 1.00 (0.25-4.00) | 1.000 | 152/369 | 0.95 (0.26-3.43) | 1.000 |
|  | *HLA-A*11:02* | 0/10 | 4/50 | 0.49 (0.02-9.86) | 1.000 | 11/369 | 1.48 (0.08-26.90) | 1.000 |
|  | *HLA-A*24:02* | 2/10 | 16/50 | 0.53 (0.10-2.79) | 0.708) | 80/369 | 0.90 (0.19-4.34) | 1.000 |
|  | *HLA-A*30:01* | 1/10 | 2/50 | 2.67 (0.22-32.61) | 0.427 | 16/369 | 2.45 (0.29-20.54) | 0.372 |
|  | ***HLA-A*33:03*** | **7/10** | **11/50** | **8.27 (1.83-37.41)** | **0.005** | **80/369** | **8.43 (2.13-33.34)** | **0.002** |
|  | *HLA-B*13:01* | 1/10 | 7/50 | 0.68 (0.74-6.25) | 1.000 | 131/986 | 0.73 (0.09-5.77) | 1.000 |
|  | *HLA-B*13:02* | 0/10 | 2/50 | 0.63 (0.03-13.75) | 1.000 | 27/986 | 1.13 (0.07-19.30) | 1.000 |
|  | ***HLA-B*15:02*** | **5/10** | **6/50** | **7.33 (1.63-33.02)** | **0.005** | **153/986** | **5.44 (1.56-19.03)** | **0.005** |
|  | *HLA-B*15:13* | 1/10 | 0/50 | 0.90 (0.73-1.11) | 0.167 | 11/986 | 9.85 (1.15-84.53) | 0.115 |
|  | *HLA-B*15:35* | 0/10 | 1/50 | 1.57 (0.06-41.32) | 1.000 | 16/986 | 2.80 (0.16-49.82) | 1.000 |
|  | *HLA-B*18:02* | 0/10 | 1/50 | 1.57 (0.06-41.32) | 1.000 | 46/986 | 0.96 (0.06-16.69) | 1.000 |
|  | ***HLA-B*35:08*** | 1/10 | 0/50 | 15.95 (0.60-421.64) | 0.167 | **1/986** | **109.44 (6.34-1889.11)** | **0.020** |
|  | *HLA-B*39:01* | 1/10 | 1/50 | 3.44 (0.31-95.21) | 0.308 | 14/986 | 7.71 (0.92-65.06) | 0.141 |
|  | *HLA-B*40:01* | 1/10 | 11/50 | 0.39 (0.05-3.46) | 0.670 | 156/986 | 0.59 (0.07-4.70) | 1.000 |
|  | ***HLA-B*44:03*** | **3/10** | **2/50** | **10.29 (1.45-72.81)** | **0.029** | **82/986** | **4.73 (1.20-18.62)** | **0.046** |
|  | *HLA-B*46:01* | 2/10 | 11/50 | 0.89 (0.16-4.79) | 1.000 | 207/986 | 0.94 (0.20-4.47) | 1.000 |
|  | *HLA-B*51:01* | 1/10 | 1/50 | 5.44 (0.31-95.21) | 0.308 | 65/986 | 1.57 (0.20-12.62) | 0.498 |
|  | *HLA-B*52:01* | 1/10 | 1/50 | 5.44 (0.31-95.21) | 0.308 | 43/986 | 2.44 (0.30-19.67) | 0.365 |
|  | *HLA-B*55:02* | 0/10 | 4/50 | 15.95 (0.60-421.64) | 1.000 | 23/986 | 1.95 (0.11-34.31) | 1.000 |
|  | *HLA-B*58:01* | 3/10 | 5/50 | 3.86 (0.75-19.84) | 0.120 | 161/986 | 2.20 (0.56-8.58) | 0.218 |
| SCAR | *HLA-A*02:01* | 0/5 | 9/50 | 0.47 (0.02-9.30) | 0.578 | 39/369 | 0.76 (0.04-14.02) | 1.000 |
|  | *HLA-A*02:06* | 0/5 | 3/50 | 1.23 (0.06-27.17) | 1.000 | 15/369 | 2.08 (0.11-39.31) | 1.000 |
|  | *HLA-A*02:07* | 2/5 | 3/50 | 10.44 (1.23-88.44) | 0.060 | 49/369 | 4.35 (0.70-26.72) | 0.139 |
|  | *HLA-A*11:01* | 3/5 | 20/50 | 2.25 (0.35-14.69) | 0.639 | 152/369 | 2.14 (0.35-12.97) | 0.653 |
|  | *HLA-A*11:02* | 1/5 | 4/50 | 2.88 (0.26-32.26) | 0.391 | 11/369 | 8.14 (0.84-78.91) | 0.151 |
|  | *HLA-A*24:02* | 2/5 | 16/50 | 1.42 (0.22-9.33) | 1.000 | 80/369 | 2.41 (0.40-14.66) | 0.302 |
|  | *HLA-A*30:01* | 0/5 | 2/50 | 1.76 (0.08-41.65) | 1.000 | 16/369 | 1.95 (0.10-36.73) | 1.000 |
|  | *HLA-A*33:03* | 0/5 | 11/50 | 0.31 (0.02-6.08) | 0.570 | 80/369 | 0.33 (0.02-5.98) | 0.589 |
|  | *HLA-B*13:01* | 1/5 | 7/50 | 1.54 (0.14-15.82) | 0.559 | 131/986 | 0.61 (0.07-5.53) | 0.511 |
|  | *HLA-B*13:02* | 0/5 | 2/50 | 1.76 (0.08-41.65) | 1.000 | 27/986 | 1.13 (0.07-19.30) | 1.000 |
|  | *HLA-B*15:02* | 1/5 | 6/50 | 1.83 (0.18-19.25) | 0.508 | 153/986 | 0.74 (0.08-6.62) | 0.571 |
|  | *HLA-B*15:13* | 0/5 | 0/50 | NA | NA | 11/986 | 7.71 (0.40-147.76) | 1.000 |
|  | *HLA-B*15:35* | 1/5 | 1/50 | 12.25 (0.64-234.81) | 0.175 | 16/986 | 0.07 (0.01-0.62) | 0.083 |
|  | *HLA-B*18:02* | 1/5 | 1/50 | 12.25 (0.64-234.81) | 0.175 | 46/986 | 0.20 (0.02-1.79) | 0.216 |
|  | *HLA-B*35:08* | 0/5 | 0/50 | NA | NA | 1/986 | 59.73 (2.18-1633.30) | 1.000 |
|  | *HLA-B*39:01* | 1/5 | 1/50 | 12.25 (0.64-234.81) | 0.175 | 14/986 | 0.06 (0.01-0.55) | 0.074 |
|  | *HLA-B*40:01* | 1/5 | 11/50 | 0.89 (0.09-8.76) | 1.000 | 156/986 | 0.75 (0.08-6.77) | 0.579 |
|  | *HLA-B*44:03* | 0/5 | 2/50 | 1.76 (0.08-41.65) | 1.000 | 82/986 | 1.00 (0.05-18.18) | 1.000 |
|  | *HLA-B*46:01* | 2/5 | 11/50 | 2.36 (0.35-15.97) | 0.582 | 207/986 | 0.40 (0.07-2.40) | 0.285 |
|  | *HLA-B*51:01* | 0/5 | 1/50 | 3.00 (0.11-82.95) | 1.000 | 65/986 | 1.28 (0.07-23.38) | 1.000 |
|  | *HLA-B*52:01* | 0/5 | 1/50 | 3.00 (0.11-82.95) | 1.000 | 43/986 | 1.97 (0.11-36.23) | 1.000 |
|  | *HLA-B*55:02* | 1/5 | 4/50 | 2.88 (0.26-32.26) | 0.391 | 23/986 | 0.10 (0.01-0.89) | 0.116 |
|  | *HLA-B*58:01* | 0/5 | 5/50 | 0.75 (0.04-15.52) | 1.000 | 161/986 | 0.46 (0.03-8.44) | 1.000 |

CI, confidence interval; CADR, cutaneous adverse drug reaction; *HLA*, human leukocyte antigen; LTG, lamotrigine; MPE, maculopapular exanthema; NA, not available; OR, odds ratio; SCAR, severe cutaneous adverse reaction; +/total, number of subjects positive for HLA allele/number of total subjects included in the study.

P-values calculated by Fisher’s exact test.

Statistically significant values are highlighted in bold.
